# Exploring the anti-diabetic potential of Australian Aboriginal and Indian Ayurvedic plant extracts using cell-based assays

**DOI:** 10.1186/s12906-015-0524-8

**Published:** 2015-02-05

**Authors:** Vandana Gulati, Pankaj Gulati, Ian H Harding, Enzo A Palombo

**Affiliations:** Department of Chemistry and Biotechnology, Faculty of Science, Engineering and Technology, Swinburne University of Technology, John Street, PO Box 218, Hawthorn, 3122 Victoria Australia

**Keywords:** Plant extracts, Anti-diabetic, Anti-cancer, Anti-oxidant

## Abstract

**Background:**

Plant-derived compounds have been used clinically to treat type 2 diabetes for many years as they also exert additional beneficial effects on various other disorders. The aim of the present study was to investigate the possible mechanism of anti-diabetic activity of twelve (seven Australian Aboriginal and five Indian Ayurvedic) plant extracts.

**Methods:**

The ethanolic plant extracts were investigated for glucose uptake and adipogenesis in murine 3T3-L1 adipocytes. Cytotoxicity studies were also carried out against two cancerous cell lines, HeLa and A549, to investigate the potential anti-cancer activities of the extracts.

**Results:**

Of the seven Australian Aboriginal plant extracts tested, only *Acacia kempeana* and *Santalum spicatum* stimulated glucose uptake in adipocytes. Among the five Indian Ayurvedic plant extracts, only *Curculigo orchioides* enhanced glucose uptake. With respect to adipogenesis, the Australian plants *Acacia tetragonophylla*, *Beyeria leshnaultii* and *Euphorbia drumondii* and the Indian plants *Pterocarpus marsupium*, *Andrographis paniculata* and *Curculigo orchioides* reduced lipid accumulation in differentiated adipocytes. Extracts of *Acacia kempeana* and *Acacia tetragonophylla* showed potent and specific activity against HeLa cells.

**Conclusions:**

The findings suggest that the plant extracts exert their anti-diabetic properties by different mechanisms, including the stimulation of glucose uptake in adipocytes, inhibition of adipogenesis or both. Apart from their anti-diabetic activities, some of the extracts have potential for the development of chemotherapeutic agents for the treatment of cervical cancer.

## Background

Type 2 diabetes has become a major health problem in both developed and developing countries. The activities of numerous plants have been evaluated and confirmed in animal models which suggest that herbal remedies could represent culturally relevant complementary and alternative treatments, as well as serve in the search for new anti-diabetic agents [1]. Readily-available high calorie foods and sedentary lifestyles are major factors for obesity which contribute to insulin resistance and type 2 diabetes. Insulin resistance is defined as defective insulin signalling and decreased insulin efficiency to induce glucose transport from the blood into key target cells. Obesity, mainly visceral fat, contributes to insulin resistance [2]. Most anti-diabetic drugs promote long-term weight gain [3]. Thus, these drugs treat one of the key symptoms, hyperglycemia, but exacerbate weight gain and obesity which further contribute to the progression of type 2 diabetes. Therefore, while these drugs are beneficial over the short-term, they are not optimal for the long-term health of type 2 diabetic patients [4]. The most desirable situation would be the development of new types of anti-diabetic drugs that are either hypoglycaemic or anti-hyperglycemic without the side effect of promoting weight gain [2]. Reducing obesity can slow down the rate of occurrence of type 2 diabetes [5]. Therefore, it is highly desirable to find new anti-diabetic agents that stimulate glucose uptake by adipose or muscle cells but, unlike thiazolidinedione or insulin, do not induce obesity or other side effects [6]. The increase in adipocyte lipid content can influence adipocyte function by reducing adiponectin secretion which promotes adipocyte differentiation, insulin sensitivity and lipid accumulation *in vivo* [7]*.* Low levels of circulating adiponectin have been linked to insulin resistance and an increased risk of diabetes. Secondary plant metabolites such as saponin glycosides, triterpenes and phenolic compounds have been reported to influence adipocyte differentiation in cultured 3T3-L1 cells, a murine fibroblast cell line that is often used as a model for adipocyte metabolism [8].

Green et al. [9] established several cloned lines of mouse 3T3 fibroblasts which are capable of differentiating into adipocyte-like cells *in vitro*. The most frequently employed adipocyte cell lines, 3T3-F442A and 3T3-L1, were clonally isolated from Swiss 3T3 cells derived from disaggregated 17 to 19-day mouse embryos. Cell lines have been used as model systems to understand various mechanisms of plants in animal and human health as they provide a continuous source of large numbers of cells necessary for proliferation and differentiation. The 3T3-L1 cell line was selected for this study because it displays relevant features including lipid storage and glucose homeostasis. During differentiation, 3T3-L1 pre-adipocytes become adipocytes with a 20-fold increase in the number of insulin receptors and acquire the ability to utilize glucose in response to insulin [10].

Many studies have exploited the Sprague–Dawley rat model (SD model) for *in vitro* evaluation of hypoglycemic activity. This is normally time-consuming, restricted to limited animal sources and involves sacrificing of animals. Therefore, the differentiated 3T3-L1 adipocyte model (3T3-L1 model) was developed as an alternative to the SD model and is used by researchers to evaluate hypoglycaemic and anti-adipogenic effects and establish the mechanisms of action. Wu et al. (2011) screened yeast extracts for hypoglycemic activity with the 3T3-L1 model, compared results with the SD model and found that the two models were highly correlated [11].

Several studies have indicated that majority of diabetic patients are obese or overweight and have higher risk of developing cancers, thus showing the association of diabetes and overall cancer incidence [12]. Cannata et al. (2010) explained hyperinsulinaemia as the mechanism linking diabetes and cancer. Insulin resistance in diabetic patients may lead to cancer by directly affecting the cancer cells via overexpression of insulin-like growth factor 1(IGF1) and insulin receptor (IR) substrate proteins [13]. The American and European Diabetes and Oncology associations published a consensus report on diabetes and cancer and agreed that most observational evidence suggests a strong link between diabetes and breast, colorectal, endometrial, liver and pancreatic cancers. The pathogenesis of the link is due to hyperinsulinaemia, hyperglycaemia, adipocytokines, growth factors, inflammation and possibly diabetes therapies [14]. Plants are rich source of phytochemicals such as carotenoids, resveratrol, quercetin, silymarin, sulphoraphane, and indole-3-carbino that protect from chronic diseases and usually target multiple cell signalling pathways [15]. Thus, we decided to explore whether Australian Aboriginal and Indian Ayurvedic plants can be utilised in the management of diabetes and related complications.

In the search for novel treatments, attention should be given to the many traditional herbal medicines for diabetes which have been employed by various ethnic groups throughout the world. One region which contains a rich flora and fauna is Australia. However, Australian Aboriginal plants have not been evaluated for their use in the treatment diabetes. Therefore, in this work, the well-characterized 3T3-L1 model was used to investigate the role of selected Australian Aboriginal and Indian Ayurvedic plant extracts for their anti-diabetic mechanisms and ability to inhibit lipid accumulation.

As all these plant extracts were previously screened for enzyme inhibition and antioxidant activity [16]. Therefore, the aim of this follow-up study was to further evaluate the anti-diabetic mechanisms of ethanolic extracts of 12 traditional medicinal plants by glucose uptake in 3T3-L1 mouse pre-adipocytes and assessing inhibition of lipid accumulation in 3T3-L1 mouse pre-adipocytes. In addition, cytotoxicity against MDCK cells, 3T3-L1 cells and human cancer cell lines (cervical carcinoma HeLa cells and lung adenocarcinoma A549 cells) was evaluated by establishing the cytotoxic concentrations of the extracts using MTT assays. The Australian Aboriginal plants were selected on the basis of availability and their known medicinal activities. The Indian Ayurvedic plants were selected according to their reported anti-diabetic potential [17]. These plants were known to possess anti-diabetic action and but not all plants had been screened using the cell-based assays used in this study. The ethno-botanical uses of the plants have been reported earlier [16].

## Methods

Dulbecco’s modified Eagle medium (DMEM), Dulbecco’s Modified Eagle Medium/Ham’s nutrient mixture F12 (DMEM/F12), fetal bovine serum (FBS), insulin, 2-[*N*-(7-Nitrobenz-2-oxa-1,3-diazol-4-yl)amino]-2-deoxy-d-glucose (2-NBDG), trypsin/EDTA and penicillin-streptomycin were purchased from Invitrogen Australia. Bovine serum albumin (BSA), 3-isobutyl-1-methylxanthine (IBMX), dexamethasone, 3-(4, 5 dimethylthiazol- 2-yl)-2, 5 diphenyltetrazolium bromide (MTT), d-biotin, rosiglitazone and Oil Red O were obtained from Sigma-Aldrich, Australia. The Madin-darby canine kidney epithelial cells (MDCK) cell line was procured from the American Type Cell Culture (ATCC). A549, HeLa and 3T3-L1 cells were provided by Monash University, Victoria, Australia. The cells were routinely passaged as described below.

### Plant extracts

Seven Australian Aboriginal medicinal plant extracts were provided by The University of South Australia, Adelaide, Australia. Powdered extracts of five Indian Ayurvedic plants were provided by Promed Research Centre, Gurgaon, India. Tables 1 and 2 shows the list of plants used in this study. Preparation of ethanolic plant extracts, voucher numbers and ethno botanical information have previously been described by our research group [16].

**Table 1:** **Australian Aboriginal plants**

**Plant name**	**Codes used**	**Common name**	**Family**	**Part used**
***Acacia kempeana*** **F. Muell.**	AK	Witchetty Bush	Mimosaceae	Leaves
***Acacia*** **.** ***tetragonophylla*** **F. Muell.**	AT	Dead finish	Mimosaceae	Stem
***Acacia ligulata*** **Cunn. ex Benth.**	AL	Umbrella bush	Mimosaceae	Leaves
***Beyeria leschenaultii*** **(DC.) Baillon**	BL	Turpentine bush	Euphorbiaceae	Leaves and stem
***Euphorbia drummondii*** **Boiss.**	ED	Caustic weed	Euphorbiaceae	Whole plant
***Santalum lanceolatum*** **R. Br.**	SL	Northern sandalwood	Santalaceae	Leaves
***Santalum spicatum*** **(R. Br.) A. DC.**	SS	Australian sandalwood	Santalaceae	Leaves

**Table 2:** **Indian Ayurvedic plants**

**Plant name**	**Codes used**	**Common name**	**Family**	**Part used**
***Andrographis paniculata*** **Nees.**	AP	Kalmegh	Acanthaceae	Herb
***Bacopa monnieri***	BM	Brahmi	Scrophulariaceae	Herb
***Curculigo orchioides*** **Gaertn.**	CO	Kali musli	Amaryllidaceae	Rhizomes
***Mucuna pruriens*** **Linn.**	MP	Konch	Fabaceae	Seeds
***Pterocarpus marsupium*** **Roxb.**	PM	Vijayasaar	Fabaceae	Wood

### Passaging of cell lines

Cells were routinely cultivated as monolayers in disposable 25 cm^2^ flasks (Corning) in DMEM supplemented with 10% (v/v) FBS, 1% (v/v), penicillin-streptomycin (10,000 U/ml penicillin and 10,000 μg/ml streptomycin in 0.85% saline) and passaged when 70-80% confluent. The medium was aspirated from the confluent cells using a sterile pipette and cells were washed with approximately 5 mL sterile 1X PBS solution, which was subsequently aspirated. Trypsin/EDTA solution (2.5 mL) was added to the flask to cover the cell monolayer and the flask was incubated at 37°C for 3 minutes to allow the cells to detach. Fresh medium (3 mL) was used to re-suspend the detached cells and neutralize the action of trypsin. The cell suspension was centrifuged at 200 g for 5 min at 20°C. The supernatant was discarded and cell pellet was re-suspended in 5 ml of fresh medium. Cell counts were carried by the trypan blue dye exclusion method. Cells were seeded at a density of 1.5 × 10^5^/flask and incubated at 37°C in 5% CO_2_ atmosphere.

### Cytotoxicity assay

All the four cell lines, 3T3-L1 pre-adipocyte, A549, HeLa and MDCK used in this assay, were capable of attachment to form a homogeneous monolayer on plastic substratum of culture wells, which is ideal for determining cytotoxicity was determined by the MTT (3-(4, 5 dimethylthiazol- 2-yl)-2, 5 diphenyltetrazolium bromide) method test. The MTT test is a simple bioassay used for the primary screening of crude plant extracts [18]. For each cell line, there was a linear relationship between cell number and absorbance; measured at 540 nm in both control and drug-treated wells. After 72 h of treatment, the IC_50_ of the plant extracts was determined. The cells were exposed to 100 μl of each test solution {containing various concentrations of plant extracts (1 – 500 μg/ml) or vincristine (0.001 – 200 μg/ml)} and incubated for a further 72 hours at 37°C. The test solutions were then removed and the cells were washed in 1X PBS and 50 μl of medium was added into each well. Then, 5 μl of MTT solution (5 mg/ml PBS) was placed into each well and incubated at 37°C. After 4 hours, 25 μl of cells were removed, 50 μl DMSO was added and the mixture incubated at 37°C for 10 min. The absorbance at 540 nm was measured using a microplate reader (Bio-Rad Laboratories).

### Adipocyte differentiation of 3T3-L1 cells

3T3-L1 cells (ATCC; CL-173) represent a subclone of the 3T3 cells which is able to undergo adipocyte differentiation. Cells were cultured and differentiated as described previously [19,20], with minor amendments. 3T3-L1 cells at passage 9 or 10 were seeded in 96-well plates (5 × 10^3^ cells/well) for Oil Red O staining and glucose uptake measurements using DMEM/F12 medium with 10% FBS. DMEM/F12 is a serum free medium which is supplemented with a defined combination of nutrients, growth factors and hormones to culture a variety of cells. Two days after reaching confluence, the medium was changed to differentiation medium (DMEM/F12 + 2% FBS containing 10 μg/mL insulin, 0.5 mM (IBMX) 3-isobutyl-1-methylxanthine and 1.0 μM dexamethasone). 3T3-L1 cells when treated with a combination of dexamethasone, isobutylmethylxanthine (IBMX) and insulin adopt a rounded phenotype and within 5 days begin to accumulate lipids intracellularly in the form of lipid droplets [21]. Cells remained in the differentiation medium for four days with media replenished every 48 hours. Thereafter, differentiation medium was replaced by DMEM/F12 + 2% FBS in which cells remained for the respective experiments.

### Glucose uptake measurements

At day 9 of differentiation, adipocytes were incubated for 24 hours with the respective test solutions. Ethanol was used as a negative control whereas 10 μM rosiglitazone was used as a positive control. Next day, the cells were rinsed with 1X PBS and incubated for 60 min at 37°C with exclusion of light in DMEM containing 80 μM of the fluorescent glucose analogue, 2-NBDG, again in the presence of the extracts for basal glucose uptake measurement. As a second positive control, cells were treated with 100 nM insulin during the 2-NBDG incubation to measure the insulin-stimulated glucose uptake. The reaction of 2-NBDG uptake was terminated by washing the cells with pre-cooled 1X PBS. The remaining fluorescence activity in the cells was measured by using fluorescence microplate reader (POLARStar Omega, BMG Labtech, Germany) at an excitation wavelength of 485 nm and an emission wavelength of 535 nm. Fluorescence activity in the absence of 2-NBDG was subtracted from all values [20].

### Lipid accumulation inhibition assay and Oil Red O staining of intracellular triglycerides

Lipid accumulation inhibition assay was carried out as per standard protocols with minor amendments [22]. 3T3-L1 cells were differentiated into adipocytes as described above. To quantify the effect of plant extracts on lipid accumulation in 3T3-L1 cells, the cells were treated with fresh plant extracts in DMEM supplemented with 2% FBS every alternate day from day 2 till day 10 of differentiation [23]. On day 10 of differentiation, the medium was removed and the cells treated with and without plant extracts were washed with 1X PBS and fixed with 10% formalin for 30 minutes. Cells were rinsed with deionized water and then incubated with Oil Red O solution (0.25% w/v in 60% isopropanol) for 1 hour at room temperature. Finally, the dye retained in the 3T3-L1 cells was eluted with isopropanol and quantified by measuring the optical absorbance at 540 nm. Cells were also imaged under a light microscope [24].

### Statistical analysis

All samples were analysed in triplicates. Data are presented as mean ± standard error mean (SEM). For the final evaluation of the glucose uptake assay, fluorescence activities measured for the negative control (solvent ethanol) were set to 100% and values for test extracts and positive controls were calculated accordingly. In the case of lipid inhibition assays, cells treated with inducers were set to 100% and values for tested extracts were calculated accordingly. Differences were evaluated by one-way analysis of variance (ANOVA) test completed by a Bonferroni’s multicomparison test. Differences were considered significant at p < 0.001. The concentration giving 50% inhibition (IC_50_) was calculated by non-linear regression with the use of GraphPad Prism Version 5.0 for Windows (GraphPad Software, San Diego, CA, USA) (www.graphpad.com). The dose–response curve was obtained by plotting the percentage inhibition versus concentration [25].

## Results

### Cytotoxicity studies

This study examined the cytotoxicity and anti-tumour activity of Australian Aboriginal and Indian Ayurvedic plant extracts. The ethanolic extracts were tested for cytotoxic effects against A549, HeLa, 3T3-L1 and MDCK cells. The cytotoxicity and selectivity of the Australian Aboriginal plant extracts against the selected cancerous cell lines are summarized in Table 3. According to the standard National Cancer Institute (NCI) criteria, crude extracts possessing an IC_50_ of <30 μg/ml are considered active against the tested cancer cells [26]. Of the seven extracts tested, only two extracts, AK and AT, showed activity according to NCI criteria with IC_50_ of 13.73 ± 1.51 μg/ml and 27.00 ± 14.28 μg/ml, respectively, against HeLa cells. Vincristine, a chemotherapeutic drug used for some cancer types, had cytotoxic effects on MDCK, A549 and HeLa with values of 145.83 μg/ml, 0.6 μg/ml and 0.39 μg/ml, respectively. The five Indian Ayurvedic plant extracts were also tested against selected leukemic cell lines. None of the extracts showed promising effects (Table 4) against the cells used in this assay.

**Table 3:** **IC**_**50**_ **values (μg/ml) of Australian Aboriginal extracts on two cancer cell lines and the non-cancerous MDCK and 3T3-L1 cell lines**

**Australian Aboriginal plant extracts/Control**		**Cell lines**		
	**3T3-L1 pre adipocytes**	**MDCK**	**A549**	**HeLa**
***Vincristine***	Not determined	145.83 ± 5.20	0.6 ± 0.02	0.039 ± 0.01
***AK***	202.36 ± 21.44	550.03 ± 36.20	75.17 ± 6.25	13.73 ± 1.51*
***AL***	240.70 ± 65.22	398.51 ± 31.26	298.6 ± 44.45	110.61 ± 35.82
***AT***	306.46 ± 65.56	567.38 ± 52.59	210.85 ± 30.82	27.00 ± 14.28*
***BL***	386.95 ± 56.32	331.33 ± 30.90	84.08 ± 24.37	229.11 ± 40.57
***ED***	256.34 ± 59.53	460.25 ± 20.96	91.26 ± 19.84	142.50 ± 29.27
***SL***	173.14 ± 24.86	406 ± 24.31	162.95 ± 27.34	186.66 ± 62.21
***SS***	158.81 ± 25.53	283.66 ± 12.64	112.28 ± 13.28	110.11 ± 11.91

Data are expressed as mean ± SEM of independent experiment (*n* = 3). *denotes IC_50_ less than 30 μg/ml which is considered as an active extract against cancer cells.

**Table 4:** **IC**_**50**_ **values (μg/ml) of Indian Ayurvedic plant extracts on two cancer cell lines, MDCK and 3T3-L1 cell line**

**Indian Ayurvedic plant extracts/control**		**Cell lines**		
	**3T3-L1 pre-adipocytes**	**MDCK**	**A549**	**HeLa**
***Vincristine***	Not determined	145.83 ± 5.20	0.6 ± 0.02	0.039 ± 0.01
**CO**	214.73 ± 42.89	403.28 ± 12.64	457.6 ± 65.93	287.61 ± 42.86
**MP**	256.34 ± 59.53	441.33 ± 59.12	301.73 ± 48.71	397.41 ± 87.83
**PM**	171.45 ± 17.06	491.95 ± 35.80	416.15 ± 108.93	380.73 ± 72.88
**AP**	352.18 ± 41.27	449.55 ± 23.13	351.31 ± 59.06	487.86 ± 47.64
**BM**	394.03 ± 25.95	540.3 ± 37.66	320.36 ± 22.08	366.05 ± 37.63

Data are expressed as mean ± SEM of independent experiment (*n* = 3).

The Australian Aboriginal plant extracts showed IC_50_ values in the in range of 158.81 – 386.95 μg/ml and Indian Ayurvedic plant extracts showed IC_50_ values in in range of 171.45 – 394.03 μg/ml against 3T3-L1 pre-adipocytes cells. Thus, two concentrations, 10 and 100 μg/ml, were selected for evaluating the effects of the extracts on adipogenesis and glucose uptake.

### Glucose uptake assay

**Figure 1:**
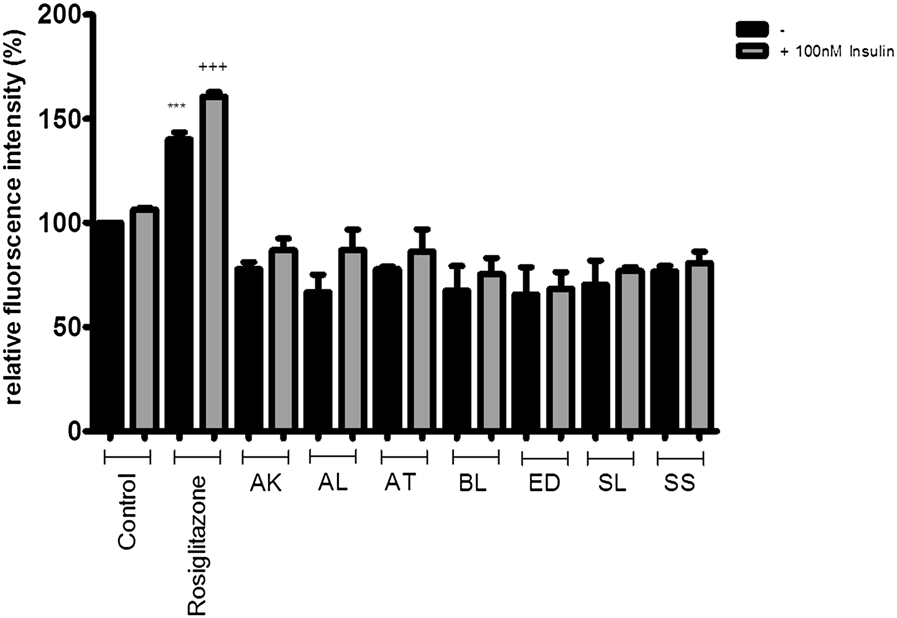
**Effect of Australian Aboriginal plant extracts at 10 μg/ml on basal and insulin-stimulated glucose uptake in 3T3-L1 adipocytes.** Cells were treated with individual extracts for 24 hours followed by incubation for 60 min in serum and glucose-free medium containing 80 μM 2-NBDG. Ethanol was used as a negative control, while rosiglitazone and insulin were used as positive controls. Cells received insulin only during 2-NBDG uptake. After incubation, fluorescence activity remaining in the cells was measured by a fluorescence microplate reader. Fluorescence activity in the absence of 2-NBDG was subtracted from all values. Data shown are mean ± SD of at least three independent experiments performed in triplicates. Significance against ethanol control (=100%): ***p < 0.001. Significance against ethanol + 100 nM insulin control: +++ p < 0.001.

**Figure 2:**
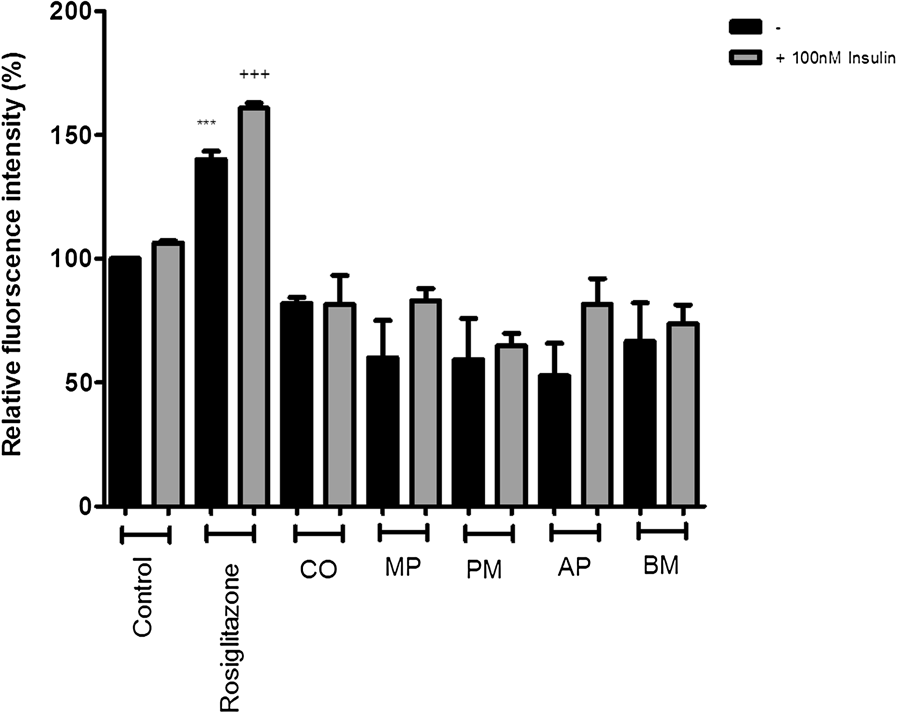
**Effect of Indian Ayurvedic plant extracts at 10 μg/ml on basal and insulin-stimulated glucose uptake in 3T3-L1 adipocytes.** Cells were treated with the individual extract for 24 hours followed by incubation for 60 min in serum and glucose-free medium containing 80 μM 2-NBDG. Ethanol was used as a negative control, while rosiglitazone and insulin were used as positive controls. Cells received insulin only during 2-NBDG uptake. After incubation, fluorescence activity remaining in the cells was measured by a fluorescence microplate reader. Fluorescence activity in the absence of 2-NBDG was subtracted from all values. Data shown are mean ± SD of at least three independent experiments performed in triplicates. Significance against ethanol control (=100%): ***p < 0.001. Significance against ethanol + 100 nM insulin control: +++ p < 0.001.

**Figure 3:**
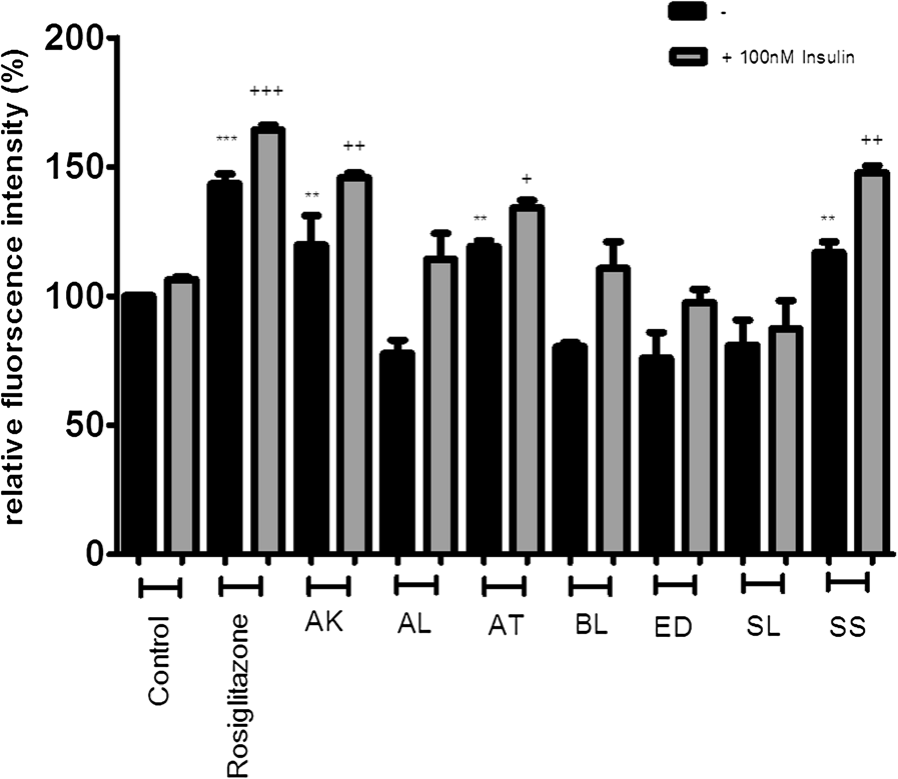
**Effect of Australian Aboriginal plant extracts at 100 μg/ml on basal and insulin-stimulated glucose uptake in 3T3-L1 adipocytes.** Cells were treated with individual extracts for 24 hours followed by incubation for 60 min in serum and glucose-free medium containing 80 μM 2-NBDG. Ethanol was used as a negative control, while rosiglitazone and insulin were used as positive controls. Cells received insulin only during 2-NBDG uptake. After incubation, fluorescence activity remaining in the cells was measured by a fluorescence microplate reader. Fluorescence activity in the absence of 2-NBDG was subtracted from all values. Data shown are mean ± SD of at least three independent experiments performed in triplicates. Significance against ethanol control (=100%): **p < 0.01, ***p < 0.001. Significance against ethanol + 100 nM insulin control: + p < 0.05, ++ p < 0.01, +++ p < 0.001.

**Figure 4:**
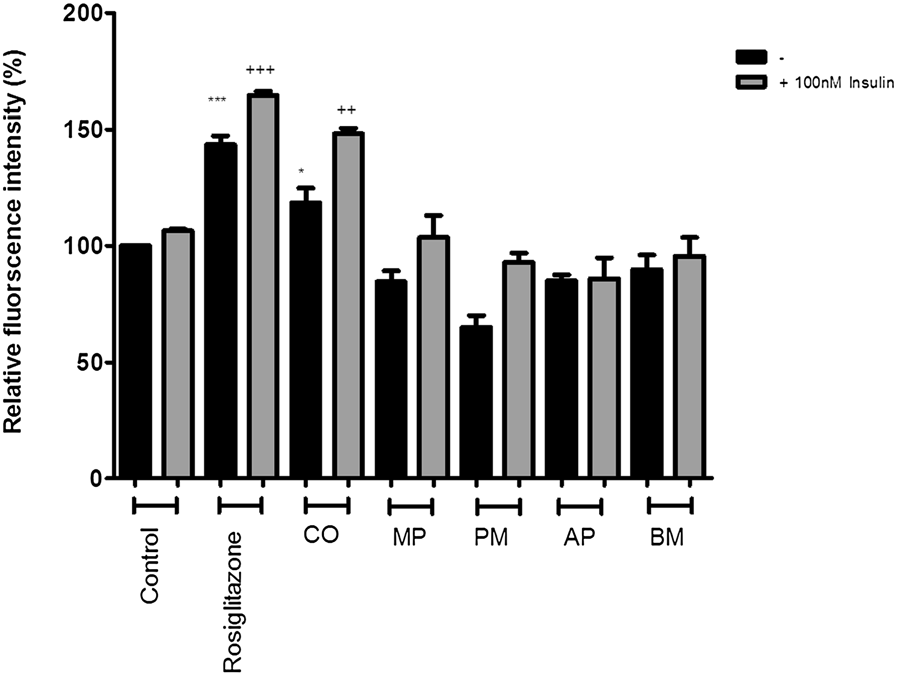
**Effect of Indian Ayurvedic plant extracts at 100 μg/ml on basal and insulin-stimulated glucose uptake in 3T3-L1 adipocytes.** Cells were treated with the individual extract for 24 hours followed by incubation for 60 min in serum and glucose-free medium containing 80 μM 2-NBDG. Ethanol was used as a negative control, while rosiglitazone and insulin were used as positive controls. Cells received insulin only during 2-NBDG uptake. After incubation, fluorescence activity remaining in the cells was measured by a fluorescence microplate reader. Fluorescence activity in the absence of 2-NBDG was subtracted from all values. Data shown are mean ± SD of at least three independent experiments performed in triplicates. Significance against ethanol control (=100%): *p < 0.05, **p < 0.01, ***p < 0.001. Significance against ethanol + 100 nM insulin control: ++ p < 0.01, +++ p < 0.001.

The seven Australian Aboriginal and five Indian Ayurvedic plant extracts were tested at 10 and 100 μg/ml concentrations to assess their impact on basal and insulin-stimulated glucose uptake into differentiated 3T3-L1 adipocytes. After incubation for 28 hours, the Australian Aboriginal (Figure 1) and Indian Ayurvedic plant extracts (Figure 2) failed to enhance basal and insulin-stimulated glucose uptake at concentration of 10 μg/ml. However, when the same extracts were tested at 100 μg/ml, it was observed that AK, AT and SS moderately enhanced basal glucose uptake by 19, 19 and 16%, respectively, as compared to the ethanol control (Figure 3). In contrast, rosiglitazone enhanced basal glucose uptake by nearly 43% as compared to control. Of the Indian Ayurvedic plant extracts tested at 100 μg/ml, only CO was able to enhance basal glucose uptake by nearly 19% as compared to control (Figure 4).

AK and SS were able to enhance insulin-stimulated glucose uptake by 45 and 47% at 100 μg/ml, respectively, whereas, the enhancement approached 65% for rosiglitazone (Figure 3) and AT enhanced glucose uptake in the presence of insulin by 34% which was approximately half that of rosiglitazone (Figure 3). CO was able to enhance glucose uptake by 48% in the presence of insulin (Figure 4).

### Inhibition of lipid accumulation in 3T3-L1 cells

**Figure 5:**
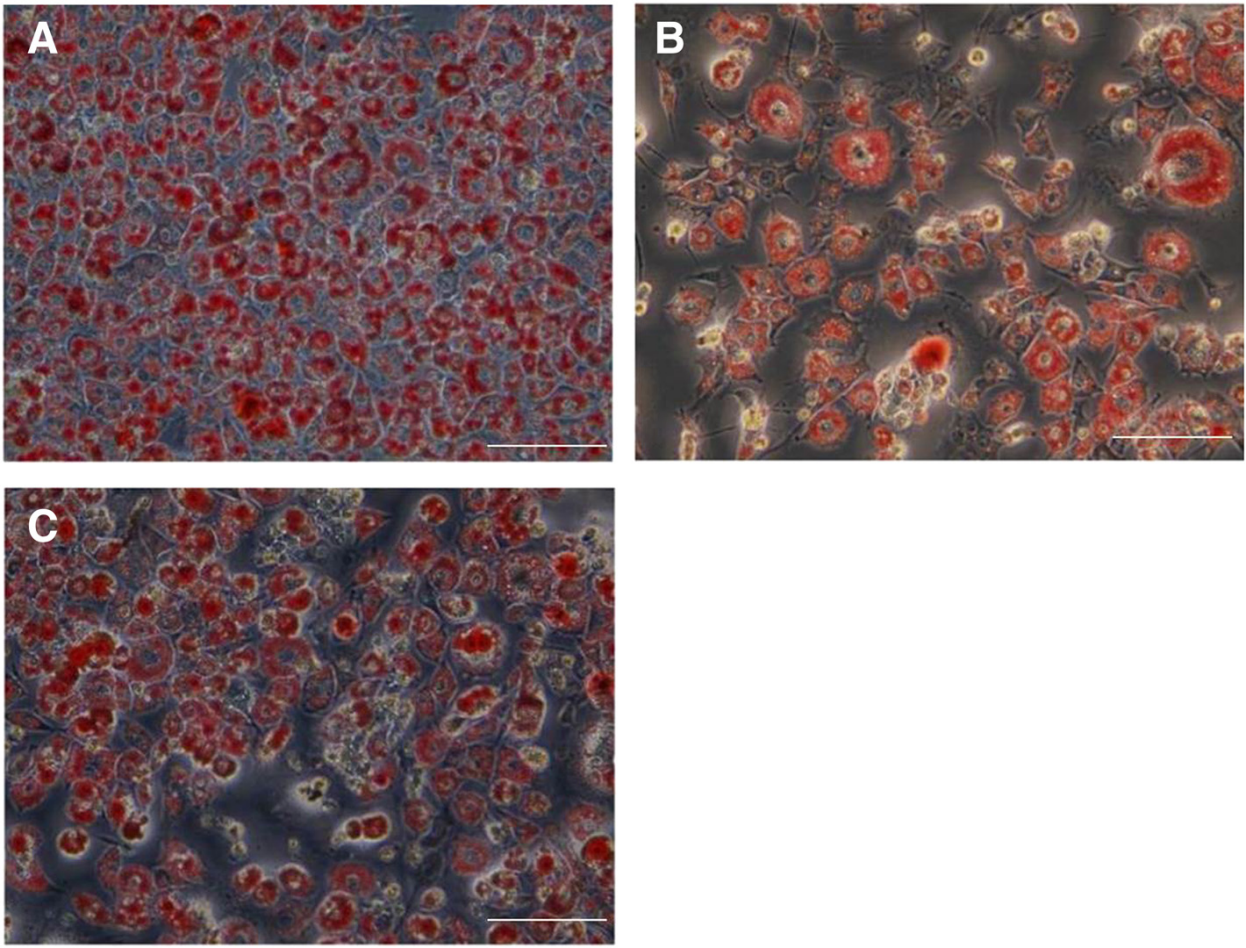
**Effect of AT (B) and CO (C) extracts on fat droplet formation in 3T3-L1 cells as compared to control (A).** Pre-adipocytes were differentiated with 100 μg/mL of AT and CO extracts treatment for 8 days after 72 hours of exposure, then stained with Oil Red O dye and examined using a light microscope. Scale bar is 50 μm.

**Figure 6:**
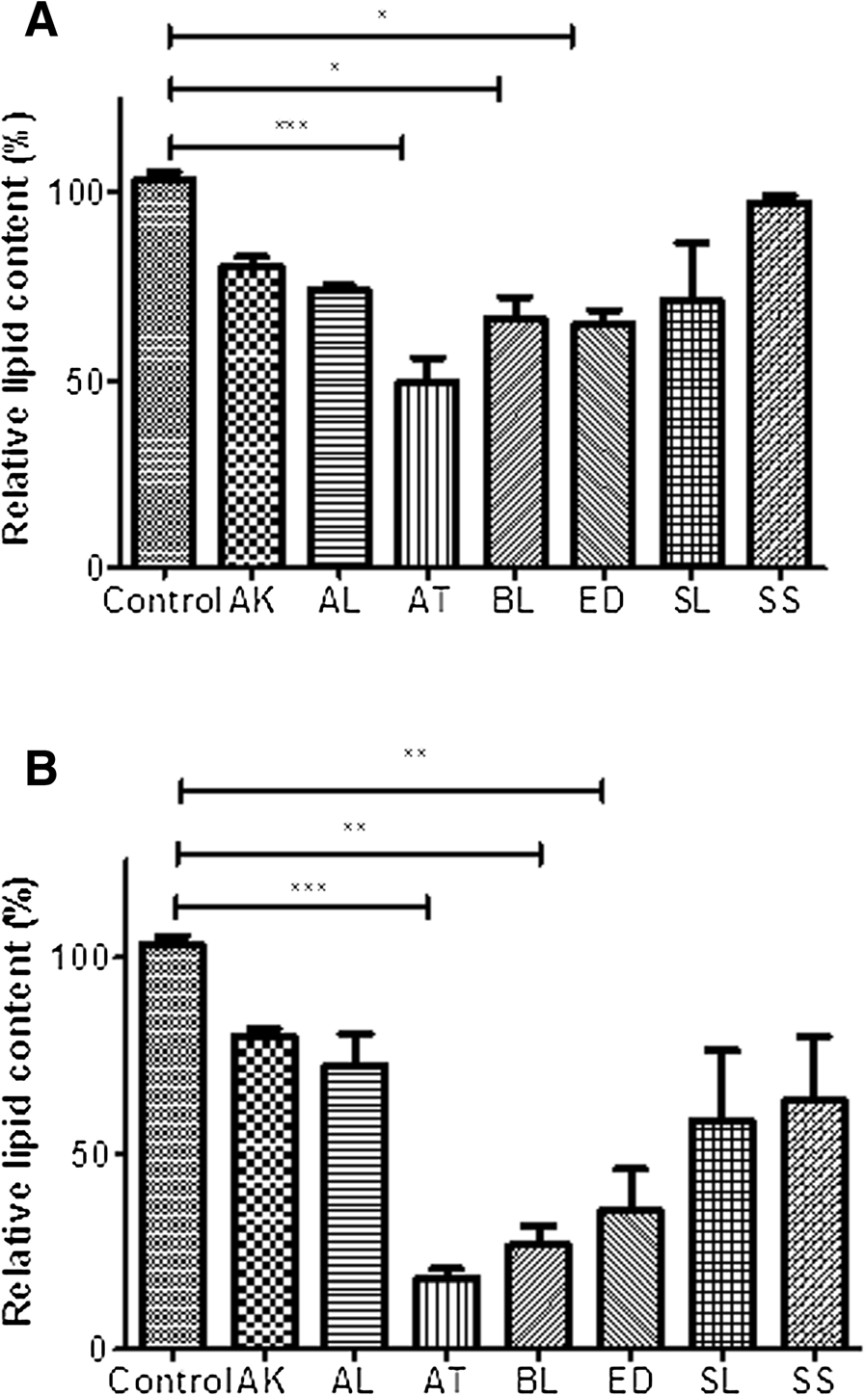
**Effect of Australian Aboriginal plant extracts on Oil Red O staining in cultured 3T3-L1 adipocytes. (A)** Effect of 10 μg/ml extracts and **(B)** Effect of 100 μg/ml extracts on fat droplet formation in 3T3-L1 cells. Values are expressed as mean ± standard deviation of at least three independent experiments. Values are mean ± SE (n = 3), significance against control (without plant extract) (=100%): ***p < 0.001 and *p < 0.05.

**Figure 7:**
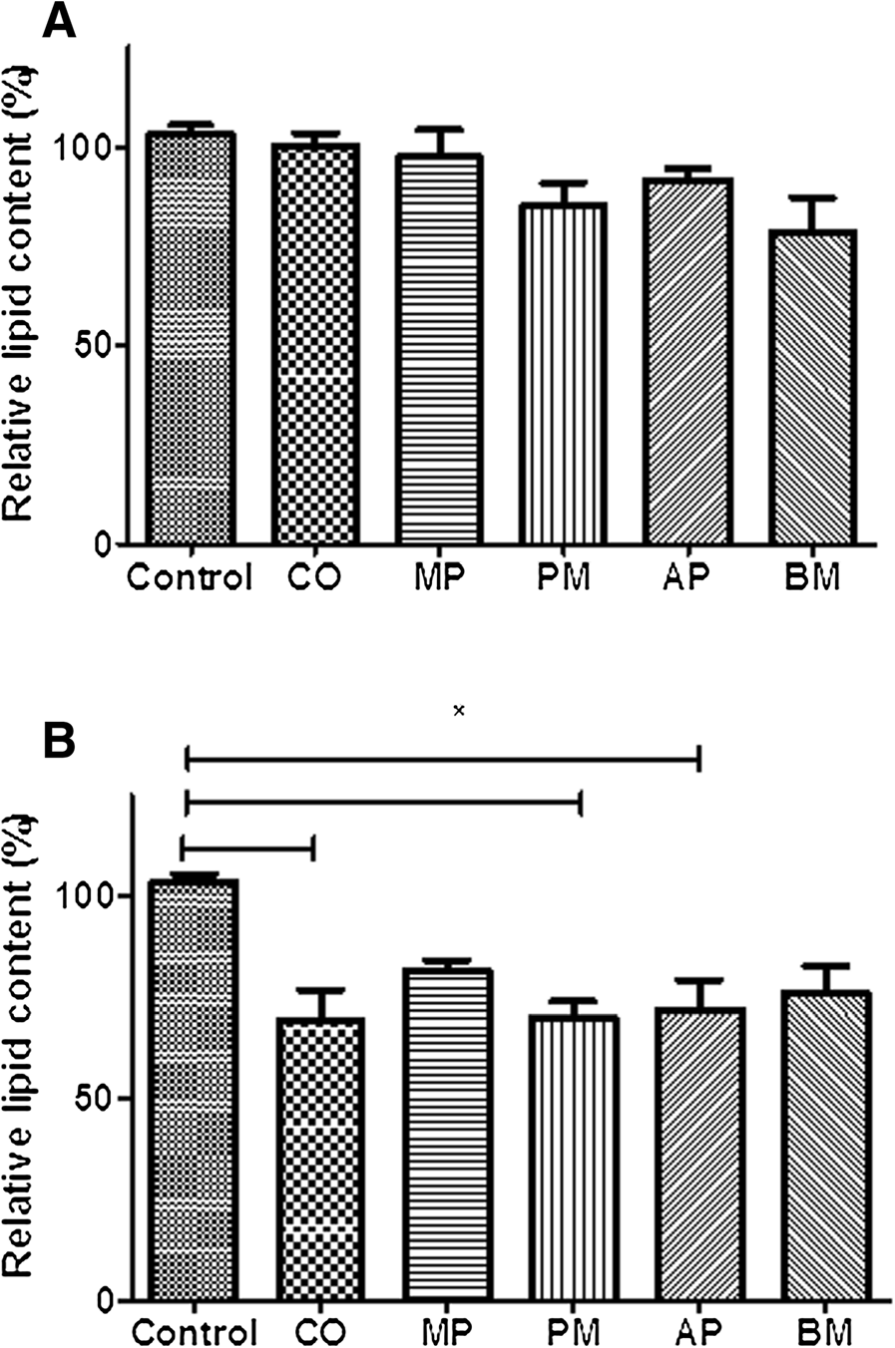
**Effect of Indian Ayurvedic plant extracts on Oil Red O staining in cultured 3T3-L1 adipocytes. (A)** Effect of 10 μg/ml extracts and **(B)** Effect of 100 μg/ml extracts on fat droplet formation in 3T3-L1 cells. Values are expressed as mean ± standard deviation of at least three independent experiments. Values are mean ± SE (n = 3), significance against control (without plant extracts) (=100%): * p < 0.05.

Adipocyte differentiation of 3T3-L1 cells is a highly-controlled process that can be induced under a hormonal cocktail of insulin, dexamethasone and IBMX [27]. Intracellular lipid accumulation is commonly monitored as a general marker to indicate the extent of adipogenesis in 3T3-L1 cells [7]. 3T3-L1 pre-adipocytes were differentiated in the presence of the Australian Aboriginal and Indian Ayurvedic plants extracts for 8 days. Figure 5 shows reduction in lipid accumulation in adipocytes treated with selected extracts. Three Australian plant extracts, AT, BL and ED, were found to significantly reduce lipid accumulation in 3T3-L1 adipocytes, suggesting anti-obesity activity. AT was able to significantly reduce lipid accumulation by 51 and 82% at 10 and 100 μg/ml, respectively (Figure 6). Lipid accumulation was reduced by 34 and 35% in presence of 10 μg/ml BL and ED, respectively, and was further reduced by 74 and 65%, respectively, with same extracts at 100 μg/ml (Figure 6). Indian Ayurvedic plants tested failed to reduce lipid accumulation at 10 μg/ml but CO, PM and AP showed moderate reduction in lipid accumulation at 100 μg/ml (Figure 7).

## Discussion

Plants have played an important role as a source of effective anti-cancer agents, and it is important to note that over 60% of the currently used anti-cancer agents are derived from natural sources, including plants, marine organisms and micro-organisms. The search for anti-cancer agents from plant sources started in the 1950s with the discovery of the alkaloids vinblastine and vincristine from *Vinca rosea* and the isolation of cytotoxic podophyllotoxins from *Podophyllum* [28]. The phytochemicals present in plants possess strong antioxidant activities that may prevent and cure cancer by protecting healthy cells from damage caused by the highly reactive oxygen species known as ‘free radicals’ [29]. Thus, consuming a diet rich in antioxidant plant foods will provide a milieu of phytochemicals that possess health protective effects, provide therapeutic actions to all cells with low cytotoxicity and are beneficial in producing nutrient repletion to immune-compromised people [30]. Strong and consistent epidemiological evidence also indicates that a diet rich in antioxidants significantly reduces the risk of many cancers [31]. Also, many studies have suggested that free radicals induce oxidative stress which leads to several disorders such as cataract, diabetes, obesity, ageing and Alzheimer’s [32].

It has been estimated that 275 Australians develop diabetes every day. The 2005 Australian AusDiab Follow-up Study (Australian Diabetes, Obesity and Lifestyle Study) showed that 1.7 million Australians have diabetes but up to half of the cases of type 2 diabetes remain undiagnosed and it is estimated that by 2033 nearly 3.5 million Australians will have type 2 diabetes [33]. Therefore, there is a great need to develop new drugs for diabetes. Part of this drug discovery research effort will be to identify plant species that can potentially be applied in the management of type 2 diabetes and related complications of weight gain, hypertension and immune-suppression. Australia is one of the mega diverse countries in the world and Australian medicinal plants are untapped source of novel chemical scaffolds and hence there is a great need to explore Australian Aboriginal plants [34].

In the present study, plants previously shown to display good antioxidant activity [16] were assessed for their cytotoxicity against cancerous (HeLa and A549) and non-cancerous (MDCK, normal epithelium and 3T3-L1 pre-adipocytes) cell lines. The cells were exposed to the extracts and the viability of cells was measured and expressed in terms of the relative absorbance of extract-treated cells, in comparison with control cells.

The results of cytotoxicity testing of Australian Aboriginal and Indian Ayurvedic plant extracts (Tables 3 and 4) were assessed according to the US NCI plant screening program, where a crude extract is generally considered to have *in vitro* cytotoxic activity if the IC_50_ value is <30 μg/ml. Among all the plant extracts screened here, two extracts, AK and AT, showed particularly potent activity with IC_50_ values of 13.73 μg/ml and 27.00 μg/ml, respectively, against HeLa cells. Other extracts showed moderate activity. None of the Indian Ayurvedic plant extracts investigated in the present study are likely candidates for anti-cancer drug development as all showed IC_50_ values of >200 μg/ml against HeLa and A549 cells. None of the Australian Aboriginal plant extracts had IC_50_ values of <30 μg/ml against A549 cells, AK, BL, ED, SS and SL had IC_50_ values of <200 μg/ml, thus they have moderate anti-cancer activity. However, only two cell lines were tested in this study and further testing against other cancer cells may reveal additional anti-cancer activity.

Based on the IC_50_ of the plant extracts, they can be divided into three groups [26]:

1.  Those with IC_50_ values of <30 μg/ml can be considered as potential candidates for further development as cancer therapeutic agents;
2.  Those with IC_50_ values between 30 and 200 μg/ml have moderate potential to be developed into cancer therapeutic agents, and;
3.  Those with IC_50_ > 200 μg/ml are unlikely candidates for development into cancer therapeutic agents.

Several studies have described that the anti-cancer activity of phytochemicals is due to their antioxidant compounds such as vitamins, minerals, polyphenols, flavonoid, terpenoids, lignins, xanthones and polysaccharides [35]. The use of natural products as medicinal agents has a long history that began with folk medicine and has been incorporated into modern medicine [36].

An important observation was that the activity against HeLa cells exhibited by extracts AK and AT was specific as no cytotoxicity was observed against the non-cancer cell line, MDCK. Therefore, these extracts may be promising candidates for the development of chemotherapeutic agents targeting cervical cancer with minimal side effects against normal cells.

The well-characterized murine pre-adipose 3T3-L1 cell line was used to investigate the mechanisms of action by which plant extracts exert their anti-diabetic effects. Since obesity is a side effect of some anti-diabetic drugs, therefore, the effect of plants on adipogenesis was also evaluated. The impact of plant extracts on basal and insulin-stimulated glucose uptake into 3T3-L1 adipocytes was examined, using the non-radioactive method of measuring 2-NBDG uptake. Of the seven Australian Aboriginal plant extracts tested, six were able to enhance insulin-stimulated glucose uptake at a concentration of 100 μg/ml. In contrast, only AK, AT and SS were able to enhance basal glucose uptake. It is well known that thiazolidinediones have beneficial effects on hyperglycemia in type 2 diabetes, but the molecular mechanism is still to be elucidated. These drugs stimulate glucose uptake either by enhancing synthesis of the insulin independent (basal) glucose transporter GLUT-1 or by increasing expression or translocation of the insulin-dependent/sensitive glucose transporter GLUT-4 [20,37].

The extracts can also be tested for their effect on protein–tyrosine phosphatase 1B (PTP1B), a cytosolic enzyme, that not only increase cellular response to insulin, but also elevates leptin signalling and are therefore, a promising strategy for the treatment of diabetes mellitus and obesity [38]. Flavonoids such as epicatechin (EC) constitute an important part of the human diet, and it can be found in green tea, grapes and especially in cocoa. EC has been reported to have anticancer activity [39] and its anti-diabetic potential can be attributed to improved insulin sensitivity [40]. Therefore, the promising findings of AT and AK extracts could be attributed to the presence of flavonoid compounds, like EC, but this needs to be validated though biochemical and HPLC-based assays. GLUT-2 transporters are known to assist in diffusion of glucose across the plasma membrane of hepatocytes and maintaining equilibrium between intracellular and extracellular glucose [41]. Therefore, extracts can be tested for their potential activity against GLUT-2 transporters in the presence of high glucose challenge in HepG2 cultured cells [42].

Phosphotyrosine (PY20) elevates upon phosphorylation of the insulin receptor and its substrate during the insulin signaling pathway for the uptake of extracellular glucose [43]. It would be useful to determine if the Australian Aboriginal plants are able to modulate PY20 expression as an increase in PY20 results in enhanced insulin binding and insulin sensitivity. Insulin-like growth factor 1 receptor (IGF-1R) is a potent activator of the phosphatidyl inositol 3 kinase (PI3K)-Akt signalling pathway and it is also an inhibitor of apoptosis or programmed cell death [44]. Hence, AT and AK extracts should be investigated further for their effect on IGF-1R and PY20 levels.

Upon the completion of adipogenesis, spindle-shaped pre-adipocytes were transformed into round-shaped cells that accumulated lipids and acquired the metabolic mechanisms to facilitate glucose uptake in response to insulin, synthesize fatty acids, accumulate triglyceride and secrete a wide variety of hormones and cytokines [7]. Therefore, intracellular lipid accumulation is commonly monitored as a general marker to indicate the extent of adipogenesis in 3T3-L1 cells [45]. The results of this study showed that the three Australian plant extracts, AT, BL and ED, were able to significantly reduce lipid accumulation in 3T3-L1 adipocytes when compared to control, suggesting anti-obesity activity which is a desirable property for an anti-diabetic drug. Though, it is not clear if the reduced lipid content is due to mechanistic perturbation, or due to increased cytotoxicity or reduced differentiation/proliferation due to long term exposure of the extracts. Therefore, these plant extracts need to be further investigated by measuring protein expression of key transcription factors like peroxisome proliferator-activated receptor gamma (PPARγ) in both *in vitro* and *in vivo* models. The extracts could also be tested for their effect on Adenosine 5′-monophosphate-activated protein kinase (AMPK) which is known to inhibit lipogenesis [46].

A number of studies have demonstrated that natural compounds like EGCG, genistein, esculetin, berberine, resveratrol, guggulsterone, capsaicin, baicalein and procyanidins inhibited adipogenesis by inhibiting preadipocyte proliferation, suppressing lipid accumulation and inducing apoptosis in mature adipocytes [47]. Pterostilbene from *Pterocarpus marsupium*, resveratrol from red grapes have been reported to activate PPAR alpha and posess glucose and lipid lowering activity [48]. Australian Aboriginal plants which are yet to be tested for their phytochemicals might be showing good activity against lipid accumulation due to presence of similar compounds like genistein, resveratrol and quercetin.

Morphological observations of cells stained with Oil Red O, a lipid stain, showed a decrease in cellular lipid content in cells treated with plant extracts. Among the Indian Ayurvedic plant extracts, CO, PM and AP (at 100 μg/ml), were able to moderately reduce lipid accumulation. DNA microarray analysis can also be looked at to understand effect of plant extracts on expression of a number of genes and long non-coding RNAs implicated to play a role in the control of adipogenesis [46].

3T3-L1 cells are widely used models of adipocyte function. *In vivo,* excessive triglyceride accumulation by the adipocyte has been linked to an increased risk of a variety of metabolic disorders [49]. Tannins, catechins and epicatechins are the most active antioxidant constituents and are found to enhance the glucose uptake and inhibit adipogenesis in differentiated adipocytes [50,51]. The presence of phenolic compounds, tannins, alkaloids, procyanidins and cyanogenic glycosides have been attributed to the hypoglycaemic action of various plants [6]. The antioxidant activity of the plants was evaluated against free radicals which can damage biomolecules in our body, cause cellular membrane peroxidation and attract various inflammatory mediators [52]. Phenolic compounds and flavonoids are known to have antidiabetic, antitumor properties, antiproliferative effects and induce apoptosis in different cancer cell lines. They are free radical scavengers, and flavonoids in particular inhibit invasion and metastasis [53].

## Conclusions

The results of the current study showed that plants extract AT probably exerts its anti-diabetic properties by stimulating glucose uptake in adipocytes with significant inhibition of adipogenesis. Plant extracts AK, SS and CO were also observed to enhance basal and insulin-stimulated glucose uptake. BL, ED, MP and PM inhibited lipid accumulation but should be further studied using anti-lipase activity assays and Western blot analysis to confirm their anti-adipogenic effect. The ability of AT to enhance glucose uptake in insulin-resistant adipocytes, in addition to its anti-adipogenic effects, suggests that this extract could be useful in the treatment of type 2 diabetes. Future studies should address the molecular mechanisms by which these plants and their active compounds regulate glucose uptake by adipose and muscle tissues. To our knowledge, this is the first study of the potential use of Australian Aboriginal plant extracts in the management of diabetes and related complications. The ability of existing therapies to target various aspects of the insulin resistance syndrome induces other metabolic abnormalities, chiefly those involved in lipid metabolism. Therefore, glucose-lowering drugs with minimal adipogenic activity are desirable and this study has demonstrated but future experiments are needed to clarify the chemical structures responsible of such biological activity.
